# The Role of mTOR in *Mycobacterium tuberculosis* Infection

**DOI:** 10.3390/biomedicines12102238

**Published:** 2024-10-01

**Authors:** Ami Patel, Lannhi Nguyen, Christina Shea, Sunjum Singh, Vishwanath Venketaraman

**Affiliations:** College of Osteopathic Medicine of the Pacific, Western University of Health Sciences, Pomona, CA 91766, USA; ami.patel@westernu.edu (A.P.); lannhi.nguyen@westernu.edu (L.N.); christina.shea@westernu.edu (C.S.); sunjum.singh@westernu.edu (S.S.)

**Keywords:** autophagy, mTOR, *Mycobacterium tuberculosis*

## Abstract

**Background/Objectives**: *Mycobacterium tuberculosis* (*M. tb*) is a pathogen that causes tuberculosis (TB), an extremely infectious disease which is responsible for millions of deaths worldwide. The severity of this pathogen is further amplified with the emergence of multidrug-resistant strains that are becoming more prevalent at an alarming rate, and novel treatments are needed. **Methods**: In this paper, we discuss the pathology *M. tb* infection. We review the literature on the role that mTOR plays in autophagy and the immune system as well as its impact on *M. tb* infection. Lastly, we discuss the current therapies targeting mTOR and potential routes to explore for future treatments. **Results**: The mTOR protein acts as a negative regulator of the autophagy pathway and presents as a potent target to establish new treatments for TB. *M. tb* survival is affected by mTOR, the PI3K/mTOR/AKT pathway, and autophagy. *M. tb* evades destruction by manipulating host cellular mechanisms, which increases resistance and complicates treatment. **Conclusions**: Targeting mTOR can enhance autophagy and increase *M. tb* clearance. Existing drugs such as everolimus, rapamycin + CC214-2, and bazedoxifene are all being currently studied for effectiveness and show positive results. Alternative therapies, including Chinese herbs, baicalin, BTLA, glutathione, and precision medicine can modulate the PI3K/mTOR/AKT pathway and the host’s immune response, resulting in increased *M. tb* clearance, and these may be the future treatments for *M. tb* infection.

## 1. Introduction

Tuberculosis (TB) caused by the bacteria *Mycobacterium tuberculosis* (*M. tb*) is one of the earliest studied infectious diseases, being discovered by Robert Koch in 1882. It is both preventable and curable; however, it remains the world’s second leading cause of death from a single infectious agent after coronavirus disease (COVID-19) [1]. According to the World Health Organization (WHO), TB was noted to be responsible for 1.30 million deaths globally in 2022, though they estimate the number to be quite more than what was reported due to COVID-related disruptions and underreporting [1]. With a death rate around 50% if left untreated, TB has the highest burden in southeast Asia and Africa, amongst other countries, primarily affecting populations affected by risk factors such as undernourishment, poverty, HIV infection, diabetes, overcrowding, lack of hygiene and smoking as stated in reports by the WHO [1]. The risk of reactivation of latent TB infection is also increased in individuals with type 2 diabetes mellitus (T2DM), which is an illness that is rapidly increasing in nations around the world, especially in areas where TB is endemic, such as India [2].

Despite a net reduction in the global TB incidence rate by 8.7% from 2015 to 2022, the sudden rise in multidrug-resistant (MDR) or rifampicin-resistant (RR) TB has increased the threat to public health and efforts to end the global TB epidemic [1]. Current treatment recommendations require a standardized regimen of drugs being administered over a lengthy 6-month course resulting in treatment success rates of 88% in drug-susceptible TB cases and 63% in MDR/RR-TB cases, as stated by the WHO [1]. However, these treatment regimens are extremely costly to manage and often have negative economic consequences for both the patients and the countries, and as a result, only 20% of MDR-TB cases are able to access them [3,4]. Individualized drug therapy has shown to have even better treatment outcomes for patients with drug-resistant TB when compared to these standardized regimens, yet it relies on drug susceptibility testing (DST) via whole genome sequencing. This would allow providers to analyze all potential resistance conferring mutations in each case of MDR-TB and tailor treatments accordingly, but it is years away from clinical implementation [5]. Furthermore, *M. tb* is continuously evolving and developing increased resistance to drugs, leading to extensively drug resistant (EDR) and eventually totally drug-resistant (TDR) TB [5]. These strains are resistant to at least Rifampin and Isoniazid (MDR-TB) as well as at least one fluoroquinolone and a second-line injectable used to treat drug-resistant TB, and it is likely that they will gain resistance to these newly implemented standardized treatment regimens in due time as well [5]. For example, following the licensing of bedaquiline and delamanid in 2014, two promising drugs for the treatment of MDR-TB, strains of *M. tb* with resistance to them both emerged in less than 2 years [5]. The rapid rate at which multidrug-resistant strains of *M. tb* are emerging highlights the dangers of tuberculosis and the heightened urgency for the continuous development of new therapies and individualized treatment regimens based on genotypic DST to curb the amplification in drug resistance. This is especially so in the current age of increased travel and migration that undoubtedly assure the spread of MDR-TB to every corner of the world.

With the BCG vaccine being the only licensed vaccine against TB, and that too only for children, it has variable efficacy and cannot completely prevent infection and transmission of TB [6,7]. Various studies have also observed the development of disseminated mycobacterial infection secondary to BCG vaccination in children, and while rare, this still poses a potential health risk and may discourage individuals from getting children vaccinated [8]. Novel therapeutic agents are needed, as 90% of individuals who develop TB each year are adults, for which the vaccine is not a reliable preventative method [1]. However, a better understanding of the pathophysiology of infection for *M. tb* will lead to the discovery of various effective mechanisms worthy of further research to serve as potential therapeutic agents in addressing the TB epidemic.

This article aims to provide a comprehensive review of the literature supporting the role of mTOR in the autophagic pathology of those infected with *M. tb.* Furthermore, understanding the direct role of mTOR in *M. tb* infection may lead to the formation of targeted therapies and better understanding of the role that autophagy plays in *M. tb* prognosis and treatment. 

## 2. Materials and Methods

To find studies for this article on the effects of mTOR on *M. tb* infection clearance, a series of steps were performed. This included collecting data on keywords, inclusion, and exclusion criteria. Information was obtained using PubMed databases and EndNote 21 version 21.3.0.20232, which is a reference management software package. Search results included terms such as “mTOR, *Mycobacterium tuberculosis*”, “mTOR Venketaraman”, “TB treatment regimens”, “mycobacterial drug targets”, “mTOR”, “mTOR, autophagy”, “mTOR autophagy, *Mycobacterium tuberculosis*”, and “drug resistant TB”. The literature search was restricted to the English language. The cited papers were published from 2002 to 2024. Attention was paid in each section to include articles that were relevant and discussed the function of mTOR and its effects on *M. tb*. Exclusion criteria included non-relevance to *M. tb* or a lack of mechanistic explanation of mTOR function.

## 3. Pathology of *M. tb*

### 3.1. Characteristics of M. tb

*M. tb* is an acid-fast, aerobic, rod-shaped, Gram-positive bacteria with a genome of about 4 Mbps. With a doubling time of 12–24 h in its optimal conditions, *M. tb* resembles Gram-negative bacteria with the presence of an outer membrane, but the similarities end there. Its asymmetric lipid bilayer consisting of an inner layer of mycolic acids and an outer layer of glycolipids and waxy components allows it to be impervious to noxious compounds and evade host immune defenses [9]. *M. tb* infection is transmitted via respiratory droplets or aerosol inhalation and primarily affects the lungs, causing pulmonary TB, but it can also disseminate to various other tissues. While *M. tb* infection can present as asymptomatic, symptomatic clinical presentations of those with active pulmonary TB infections include malaise, persistent cough with or without purulent/blood-stained sputum, chest pain, hemoptysis, fever, weight loss, or nights sweats [7,10]. Extrapulmonary TB symptoms for those with disseminated *M. tb* infection include, but are not limited to, pleuritic chest pain, lymphadenopathy, or meningitis occurring through lymphohematogenous dissemination [7].

### 3.2. Acute M. tb Infection

When the *M. tb* bacilli are dispersed into the air via respiratory droplets from a patient with active pulmonary TB, they can then be inhaled by a new host and deposited into the alveoli. The bacilli are then quickly phagocytized by alveolar macrophages that can then eliminate the invading pathogen as a result of the host’s innate immune response. If *M. tb* is able to evade this initial host defense mechanism, it will begin to actively replicate in macrophages and express a variety of key mediators that play a role in manipulating the immune response and aid in its proliferation. By evading phagosomal elimination via these key mediators, the *M. tb* bacilli can then translocate into the cytoplasm, escaping from the phagosome with the help of early secreted antigenic target 6 kDa (ESAT-6) and cytosolic phospholipase A2 (cPLA2) virulence factors [11]. Virulence factor ESAT-6 aids *M. tb* by disrupting host cell macrophage activation and inducing apoptosis, resulting in abnormal activation of nuclear factor κB (NF-κB) and improper expression of NF-κB-dependent genes, which promote pathogen survival [12]. The bacilli can further diffuse into nearby epithelial and endothelial cells or other organs through hematogenous dissemination and the lymphatic system in the early stages of infection. The toll-like receptors (TLRs), also known as pattern recognition receptors (PRRs), expressed by macrophages recognize and bind *M. tb* ligands in the host environment, inducing dimerization of the TLRs and activate downstream signaling to increase the production of several pro-inflammatory cytokines to eliminate *M. tb*. This signaling cascade eventually results in the phosphorylation of the inhibitor of nuclear factor κB kinase (IkB) via its kinase complex (IKK), which will activate NF-κB and increase downstream interferon-γ (IFN-γ) synthesis. This will promote an inflammatory response and generate reactive oxygen species (ROS) via NADPH oxidase 2 (NOX2) [13]. The activation of the cell-mediated immune response via the recognition of *M. tb* antigens by antigen-presenting cells (APCs) recruits CD4+ T cells to migrate to the lungs and initiate granuloma formation to trap the *M. tb* bacilli and reduce bacterial replication, leading to latent TB infection [14].

### 3.3. Latent M. tb Infection

Granuloma formation is a key characteristic of *M. tb* infection and is best described as an organized collection of various immune cells such as macrophages, multinucleated giant cells, lymphocytes, neutrophils, and fibroblasts. It allows these immune cells to enact a localized inflammatory response rather than a systemic one, encasing the bacilli within to prevent further dissemination, and inhibiting *M. tb* replication within its caseous center. While found in both acute and latent infections, it is suspected that latent TB granulomas consist of both lymphocytes and macrophages, while acute TB granulomas consist primarily of macrophages [15]. Granuloma formation is significantly dependent on the production of the pro-inflammatory cytokines IFN-γ and tumor necrosis factor-alpha (TNF-α) from T cells, as studies have shown an increased susceptibility to TB and loss of granuloma structure in humans treated with TNF-α inhibitors [14,16]. While this process is primarily driven by T cells and macrophages, there is new evidence suggesting B cells and the humoral immune response also play a vital role in granuloma formation, but more research is needed to discern the mechanism behind this [14]. While most individuals develop granulomas that can contain and clear the pathogen in response to *M. tb* infection, in TB patients, *M. tb* is able to manipulate granulomas into supporting bacterial growth and dissemination using virulence factor ESAT-6 with its mitotoxic effects and the RD1 locus, which encodes a specialized secretion system that enhances infection within the granuloma [17,18]. The caseous and necrotic center of the granuloma is a result of hypoxic conditions that enhance bactericidal mechanisms such as increasing levels of ROS and apoptosis or infected cell death [19]. When activated via the innate immune response and hypoxic conditions of the granuloma, the alveolar macrophages produce ROS and nitric oxide (NO) to enhance the host antibacterial defenses and kill the invading bacilli [20]. Necrotic pathology in *M. tb* infection is in part also due to ferroptosis, known as iron-dependent lipid peroxidation-mediated cell death, due to the accumulation and uncontrolled production of ROS. To counter this excess damage to host tissues, a set of antioxidant pathways involving glutathione (GSH) and glutathione peroxidase-4 (Gpx4) keeps ROS levels in check. Gpx4 has been shown to play a critical role in curbing the severity of *M. tb* infection and necrosis by regulating the oxidative stress and decreasing the bacterial load of *M. tb*, while deficiencies in Gpx4 lead to increased lung necrosis and bacterial burdens [21].

As previously mentioned, the production of ROS and NO is a pro-inflammatory process induced by the activation of NF-κB. It plays a multifaceted role in this process, most notably by upregulating the production of membrane transport molecules in order to enhance phagolysosome fusion during an infection. The optimal activation of NF-κB is required to contain a granuloma and decrease bacterial load, as increased activation can lead to a diminished T cell response, and decreased activation was shown to increase *M. tb* growth, highlighting the therapeutic potential of an optimal signaling strength of NF-κB [20]. Despite the bactericidal effects of the hypoxic state within the granuloma, *M. tb* are able to sense the onset of these harsh conditions of low oxygen and nutrient depletion and enter a dormant state in which they continue to be metabolically active but not replicating and thus entering the latent stage of infection [9]. Latent *M. tb* can then deploy a number of virulence mechanisms to enhance survival and diffuse to surrounding cells and reactivate to persist in various organs and tissues when it encounters non-hypoxic environments. Reactivation can also occur in response to other factors such as loss of microbial diversity, excess glucocorticoid use, or other conditions affecting host T cell function [22].

In summary, the mechanism of action for both acute and latent *M. tb* are full of complexities both known and unknown, and with the use of further research, they provide various avenues for future therapeutic targets in eradicating the global TB epidemic. We will be focusing further on the specific mechanisms at play surrounding mTOR and how they can be manipulated in respect to autophagy.

## 4. Role of mTOR in the Immune System

### 4.1. Mechanism of Action of mTOR

**Figure 1:**
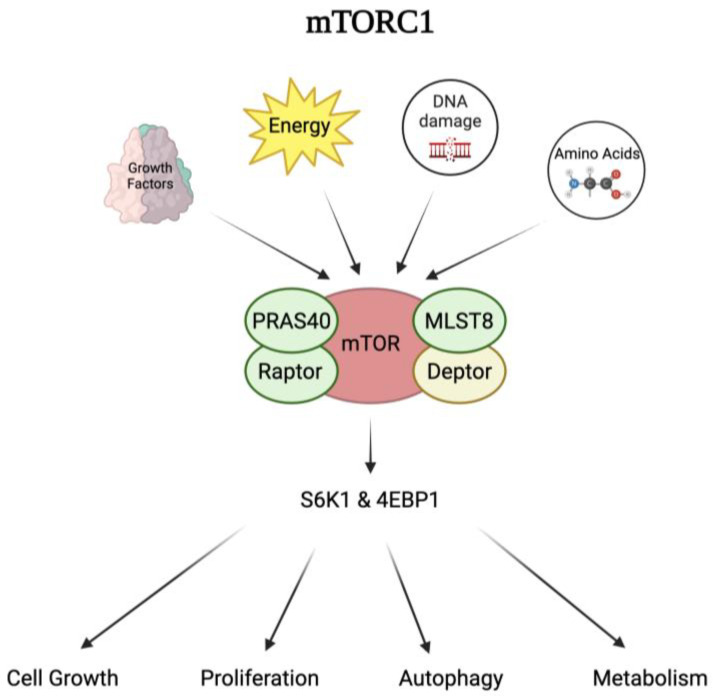
An overview of mTORC1 signaling. The mTORC1 dimer consists of 5 proteins: mTOR catalytic subunit, Raptor, PRAS40, mLST8, and Deptor. This complex receives signals from growth factors, energy levels, DNA damage, and amino acids which can activate or inhibit the effector proteins S6K1 and 4EBP1. These can go on to regulate cellular processes such as cell growth, proliferation, autophagy, and metabolism. Abbreviations: mTORC1: mTOR complex 1; Raptor: regulatory-associated protein of mTOR; PRAS40: proline-rich AKT substrate 40 kDa; mLST8: mammalian lethal with Sec13 protein 8; Deptor: DEP-domain-containing mTOR-interacting protein; S6K1: ribosomal protein S6 kinase 1; 4EBP1: eukaryotic initiation factor 4E-binding protein 1.

*M. tb* infection can activate the mammalian target of rapamycin, mTOR. mTOR is a serine-threonine kinase that forms two complexes including mTOR complex 1 (mTORC1) and mTOR complex 2 (mTORC2), which are differentiated by their activator proteins, Raptor and Rictor, respectively [23]. mTORC1 is a dimer composed of five different proteins including the mTOR catalytic subunit, the regulatory-associated protein of mTOR (Raptor), mammalian lethal with Sec13 protein 8 (mLST8), proline-rich AKT substrate 40 kDa (PRAS40), and DEP-domain-containing mTOR-interacting protein (Deptor) [24]. The complex is a central regulator for many cellular processes including cell growth, proliferation, metabolism, autophagy, and survival. It integrates complex signals from growth factors, energy levels, amino acids, and DNA damage to regulate and control the downstream cellular processes of protein synthesis, cell growth, and proliferation [23], Figure 1.

Although the functions of each protein are still unclear, it is proposed that each subunit plays a role in either activating or inhibiting mTORC1. For example, Raptor plays an important role in sensing nutrient and growth factor signals, functioning as a scaffolding protein that recruits substrates to bind and activate mTORC1 [25]. Once mTORC1 is activated, the complex can activate or inhibit downstream effector proteins, such as p70 ribosomal protein S6 kinase 1 (S6K1) and eukaryotic initiation factor 4E-binding protein 1 (4E-BP1), to induce cell regulation. In contrast to Raptor, PRAS40 and Deptor play an inhibitory role in mTORC1 activity when dephosphorylated. When PRAS40 and Deptor are phosphorylated, their affinity for mTORC1 is reduced, weakening their inhibitory effects and allowing cell growth and survival [26,27]. The exact role of mLST8 in mTOR activation is unclear [24].

mTORC1 is typically regulated at the lysosomal membrane, where Rheb (Ras homolog enriched in brain), a small GTPase, is located. Nearby is the tuberous sclerosis complex 1/2 (TSC 1/2), which is an important sensor for mTORC1 activity. TSC1/2 is a heterodimer that consists of TSC 1 and TSC 2. Rheb is activated when it is bound to GTP, which then stimulates mTORC1 [24]. TSC1/2 functions as a GTPase-activating protein (GAP) by converting Rheb into its inactive GDP-bound form [28,29]. Several different regulators can affect TSC1/2. There is an inverse control of TSC1/2 on mTORC1, where the inhibition of TSC1/2 will ultimately activate mTORC1. Growth factors stimulate insulin and Ras signaling pathways which inhibit TSC1/2 GAP function through AKT and ERK1/2, respectively. In contrast, DNA damage or low energy levels will activate p53 or AMPK pathways to stimulate TSC1/2 and decrease mTORC1 activity. Not only can mTORC1 be regulated at the upstream TSC1/2 and Rheb site, but its activity can also be modulated at the Rag GTPase site where the presence of amino acids can affect mTORC1 activity. For example, AMPK (adenosine monophosphate-activated protein kinase) serves as an energy sensor, detecting a low ATP/ADP ratio and phosphorylating TSC2, which inhibits mTORC1 activity [23,24]. Hypoxia-induced ATP depletion can also inhibit mTORC1 through the AMPK sensor [24]. Further, DNA damage can cause TSC1/2 deficiency, which can lead to increased cell death by increasing mTORC1 activation [30]. There are different proposed mechanisms involved including p53 activation of AMPK to regulate TSC1/2 and suppress mTORC1 [24].

**Figure 2:**
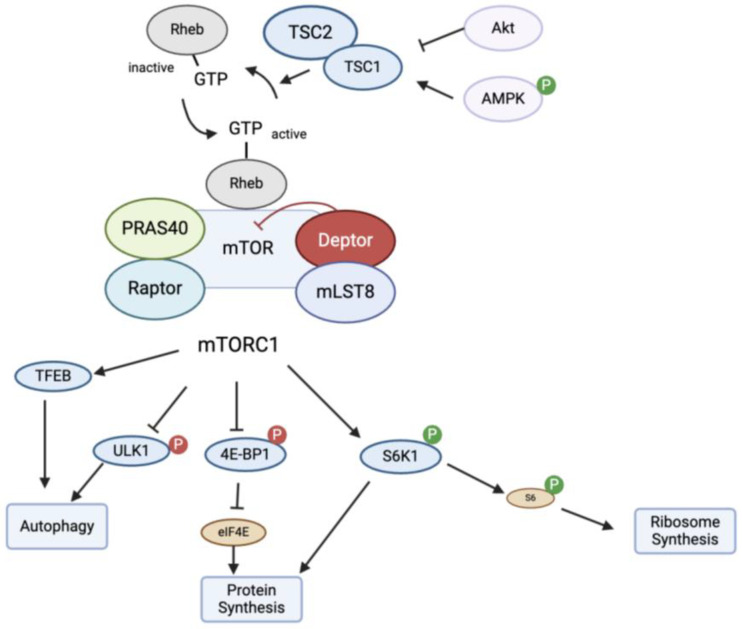
When the host is infected by *M. tb*, the body responds by activating the mTOR pathway, which involves both mTORC1 and mTORC2. mTORC1 is a key regulator for cell growth and metabolism. Protein synthesis can be activated by the phosphorylation of proteins such as S6K1 and 4E-BP1. mTORC1 inhibition through the ULK1 pathway can lead to increased autophagy. Factors including TSC1/2 at the lysosomal membrane can regulate the activity of mTOR through Rheb GTPase. Abbreviations: mTORC1: mammalian target of rapamycin complex 1; TSC: tuberous sclerosis complex; AMPK: adenosine monophosphate-activated protein kinase; PRAS40: proline-rich AKT substrate 40 kDa; mLST8: mammalian lethal with SEC13 protein 8; TFEB: transcription factor EB; ULK1: unc-51-like kinase 1; 4E-BP1: eukaryotic initiation factor 4E-binding protein 1; S6K1: p70 ribosomal protein S6 kinase 1; eIF4e: eukaryotic initiation factor 4E.

Once mTORC1 is activated, it can induce the phosphorylation of several different enzymes and ultimately increase protein synthesis, encourage cell growth, and regulate mitochondrial metabolism. mTORC1 can phosphorylate and activate p70-S6 kinase (S6K1), which then phosphorylates S6. The phosphorylation of S6 leads to an upregulation of protein translation and induces cell growth. During nutrient starvation or rapamycin treatment, the phosphorylation of S6K1 and S6 is reduced, thus causing a lack of mTOR signaling and inhibition of the formation of autophagosomes [25,31]. mTORC1 activation can also lead to the phosphorylation of 4E-BP1, which inhibits its binding to eukaryotic initiation factor 4E (eIF4E) and promotes protein synthesis [23,29,32]. In contrast, if mTORC1 is inhibited, cellular autophagy is increased through the ULK1 pathway. With various input signals, mTOR can have many different downstream effects on protein synthesis, metabolism, and autophagy (Figure 2).

### 4.2. mTOR in Autophagy

Autophagy is a cellular mechanism in which damaged organelles and protein aggregates are removed through lysosomal degradation. This process allows the cell to survive during stressful conditions and removes pathogens during infection. The formation of an autophagosome is the beginning of autophagy. The autophagosome is a double-membrane bound vesicle which becomes degradative once it contacts a lysosome, forming an autolysosome [33]. PI3K, ULK1, and autophagy-related complex ATG are all involved in the induction of autophagy, and it is powerfully induced by starvation, rapamycin, and intracellular infection [34,35]. Regarding *M. tb* infection, autophagy plays an important antimicrobial role by decreasing bacterial growth and inflammation [34,36]. In this review, we researched various papers studying the mTOR mechanism’s role in autophagy generally and during *M. tb* infection.

**Figure 3:**
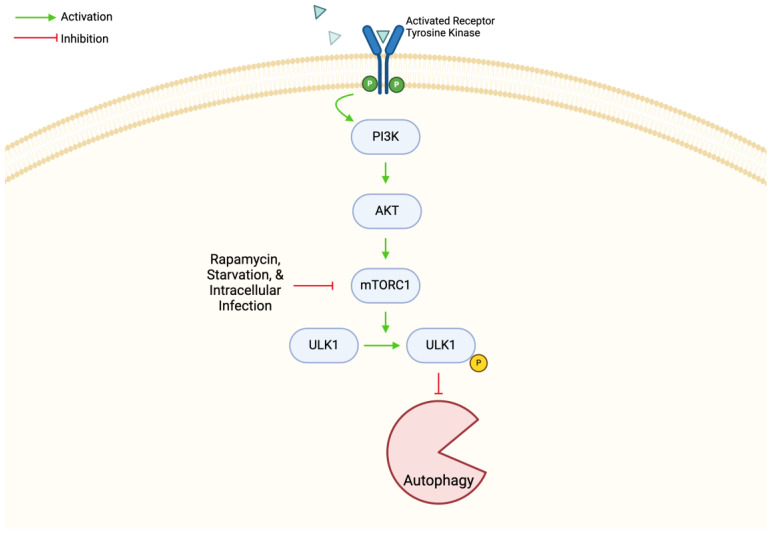
**mTORC1 is regulated by the constitutively active PI3K/AKT pathway. mTORC1 will normally phosphorylate ULK1, which will suppress autophagy**. Rapamycin, starvation and intracellular infection can inhibit mTORC1, leading to a downstream induction of autophagy. Thus, the PI3K/AKT pathway and mTORC1 are possible targets for the regulation of autophagy. Abbreviations: mTORC1: mammalian target of rapamycin complex 1; PI3K: phosphoinositide 3-kinase; AKT: protein kinase B; ULK1: UNC-51-like kinase 1.

mTORC1, one of two mTOR complexes, is regulated by the constitutively active PI3K/AKT pathway to suppress autophagy (Figure 3). *M. tb* and its components can trigger the pathway in macrophages, while rapamycin can suppress mTORC1 and activate autophagy, leading to an antimicrobial effect on *M. tb* replication. There are multiple studies showing that inhibiting the mTOR/AKT pathway is promising against cancer and *M. tb* via the regulation of autophagy [37]. As previously discussed, TSC is a very important sensor for regulating mTORC1. In Pan et al., they studied how the sustained activation of mTORC1 affected autophagy in TSC1 KO macrophages. It was found that sustained mTORC1 activation in TSC1-deficient macrophages caused an accumulation of autophagic markers and increased autophagosome formation [35]. They concluded this increase in autophagy was through the AMPK-dependent regulation of ULK1. This autophagic increase also caused a suppression in inflammation during mycobacterial infection [35]. These findings were the opposite of what the researchers originally hypothesized, showing that autophagy can occur from a different pathway but still within the same machinery. These results present another example of how mTOR and its impact on autophagy can affect *M. tb* survival in the host. However, targeting mTORC1 as treatment for *M. tb* is not always beneficial. In HIV coinfected cells, it was found that the inhibition of mTORC1 led to an increase in *M. tb* replication. These data suggested that *M. tb* can control autophagy by activating mTORC1 and thus quelling auto-phagophore formation. In addition, phagosomal maturation in HIV coinfected human monocyte derived macrophages (hMDMs) decreases when mTORC1 is inhibited. This effect explains why there is more sensitivity toward mTOR inhibition and results in increased *M. tb* growth. It was also found that in a controlled infection setting, the inhibition of mTOR resulted in a dose-dependent increase in *M. tb* growth. This was particularly pronounced in HIV-coinfected hMDMs. In this case, the inhibition of mTOR caused a loss of control of *M. tb* growth [38]. mTORC1 is yet another aspect of mTOR which can be manipulated by *M. tb* to increase its survival in the host via the regulation of autophagy. Therefore, it presents as an additional example of mTOR’s essential nature in autophagy and mycobacterial mechanism of action.

The mTOR complex is a crucial negative regulator of autophagy and is activated in the host when nutrients are available but inactivated during states of starvation [33]. Due to its importance in regulating autophagy, the mTOR pathway is widely studied as a target for therapeutic avenues in cancers and *M. tb* infections. One study showed that *M. tb* proteins inhibited autophagy via increasing mTOR signaling in macrophages during *Mycobacterium smegmatis* infection. The findings also suggested that the proteins might be activated at various points during infection to control autophagy by regulating the mTOR pathway [39]. This provides valuable insight into the importance of autophagy regulation via mTOR during mycobacterial infection.

The PI3K/AKT/mTOR pathway has been shown to regulate the autophagy process with mTOR being the central negative regulation checkpoint. Studies have shown that this pathway can be targeted to increase drug sensitivity and antitumor therapy [40]. Some Chinese herbal plants such as curcumin or larrea tridentata downregulate the mTOR/PI3K/AKT pathway, therefore upregulating autophagy and promoting antimycobacterial effects [34]. Baicalin is a flavonoid which also inhibits the same pathway to activate autophagy and kill intracellular *M. tb* [34,36]. While the mechanism may be unclear, B and T lymphocyte attenuator (BTLA) has been shown to influence the elimination of *M. tb* by enhancing autophagy through the PI3K/AKT pathway in macrophages. Silencing BTLA caused AKT and mTOR phosphorylation to increase, suggesting that BTLA potentially suppresses the AKT/mTOR pathway to trigger autophagy in mycobacterial clearance [41]. Another cellular structure which acts on the PI3K/AKT/mTOR pathway is heparin-binding hemagglutinin (HBHA). HBHA is a mycobacterial surface antigen required to bind epithelial cells and has been shown to inhibit autophagy. It promotes p-mTOR, p-PI3K, and p-AKT expression levels by activating the pathway to suppress macrophage autophagy, indicating mTOR signaling contributes to the HBHA-mediated inhibition of autophagy. This increases intracellular bacterial survival and enhances mycobacterium immune evasion [42]. In summary, multiple studies have demonstrated this pathway plays a major role in the regulation of autophagy and thus affects *M. tb* clearance. This further supports the idea that mTOR is a valuable protein of study for autophagy and *M. tb* infection.

## 5. Interplay between mTOR and *M. tb* Infection

The mTOR signaling pathway regulates many cellular processes including cytokine production, macrophage activation, and metabolic regulation. Alterations within the mTOR pathway can impact both host immune responses and metabolic adaptations to infection [43]. To survive, *M. tb* utilizes many different host immune system evasion tactics including manipulation of the mTOR signaling pathway.

Utilizing virulence factors is one of the most important mechanisms for *M. tb* to circumvent macrophage apoptosis and autophagy. Embedded within the cell wall of *M. tb* is a glycolipid called sulfolipid-1 (SL-1) that acts through the mTORC1–TFEB axis [44]. SL-1 inhibits mTOR complex 1 (mTORC1) activity, leading to the nuclear translocation of transcription factor EB (TFEB), and therefore allows lysosomal biogenesis and arrests lysosomal maturation within macrophages [44,45]. Similarly, the PI3K/AKT/mTOR pathway, through the proposed mechanism of exploiting host macrophage-derived interleukin-16 (IL-16), also helps inhibit normal phagosome maturation [46]. This arrest in maturation presents lysosomal fusion to phagosomes, revealing how *M. tb*-infected macrophages are associated with elevated levels of lysosomal content and decreased activity [47]. Normally, lysosomes contain hydrolytic enzymes and fuse with phagosomes to eliminate products brought by autophagy and phagocytosis. However, due to the prevention or delay of phagolysosomal formation, *M. tb* has time to evade destruction and promote its replication [46]. The ineffective maturation and formation of phagolysosome also allows *M. tb* to survive and escape the phagosome into the cytosol [48]. Another proposed mechanism that inhibits phagolysosome maturation is through virulence factor ESAT-6, which ultimately prevents autophagy [49]. *M. tb* can manipulate host lysosome and phagosome homeostasis through different virulence factors to circumvent the immune system and enhance its survival within cells.

Not only does ESAT-6 prevent autophagy through inhibition of phagolysosome fusion, but it can also increase inflammation within the host. Because ESAT-6 increases lysosomal permeability, an *M. tb* infection can cause the lysosome secretion of cytokines, such as IL-1β [47]. Although IL-1β is known to defend the host against *M. tb*, excessive production of the cytokine increases immunopathology [50]. To further contribute to the immunopathology, *M. tb* proteins can induce the macrophage and monocyte release of anti-inflammatory cytokines, such as IL-10 and TNF-a, or interfere with the presentation of the *M. tb* antigen to T cells, which decreases T cell activation [49,51]. During chronic infection of *M. tb*, the PI3k/AKT/mTOR signaling pathway can be suppressed, leading to an increased differentiation of T-lymphocytes into FoxP3+T lymphocytes (Treg) [49,52]. This dampens the host immune response to infection, aiding *M. tb* in its evasion strategies and prolonging infection.

Other virulence factors utilized by *M. tb* to evade host immune responses by manipulating autophagy include RipA, MoxR1, enhanced intracellular survival (EIS), and mycobacterial acyl carrier protein (AcpM). The *M. tb* endopeptidase, RipA, increases the activation of the PI3K/mTOR/AKT signaling pathway and inhibition of autophagy initiation kinase ULK1 via phosphorylation in a Toll-like receptor 4 (TLR4)-dependent manner, ultimately inhibiting autophagy and allowing the *M. tb* to evade macrophage clearance [13]. Increased mTOR activation inhibits autophagy by inhibiting ULK1 via phosphorylation. Additionally, MoxR1, an ATP-dependent chaperone produced by *M. tb*, is responsible for secreting RipA and therefore is also a regulator of host autophagy [53]. The EIS protein is another protein secreted by *M. tb* to promote its survival in macrophages by increasing its persistence and drug tolerance via the inhibition of autophagy, cell death, and inflammation by JNK-dependent inhibition of ROS generation [54]. EIS increases mTOR activity in an IL-10-dependent manner and can be neutralized by the inhibition of IL-10 production [54]. Lastly, AcpM has been shown to significantly decrease mycobacterial clearance by upregulating microRNA-155-5p expression, an SHIP1-targeting miRNA, which then increases activation of the AKT/mTOR pathway and inhibits phagolysosomal fusion via a currently unknown mechanism [55].

The initial host immune response to *M. tb* can be organized into two phases with a distinct pattern of metabolism. Upon initial infection, pro-inflammatory cytokines are released and correlate with a metabolic shift. TLR4 agonist LPS stimulate macrophages to shift from oxidative phosphorylation (OXPHOS) to glycolysis. Alterations to the glucose metabolism cause an increase in tricarboxylic acid cycle (TCA) intermediates, leading to stabilized hypoxia inducible factor-α (HIF-1α) and ultimately resulting in the transcription and production of pro-inflammatory IL-1β [56,57]. As infection progresses, activation of the mTOR/AKT pathway can impair glycolysis and mitochondrial metabolism in macrophages, making them more susceptible to *M. tb* cytotoxicity [17,52]. The signaling pathway mediates the host macrophage switch from a pro-inflammatory state to an anti-inflammatory state, which is marked by an energy shift from glycolysis to OXPHOS. This favors fatty acid metabolism, which provides a carbon source for *M. tb* [56]. These signaling pathway shifts, and the accompanying metabolic changes, allow *M. tb* to survive and persist within the host. 

Additionally, program cell death 4 (PDCD4) is a protein linked to immune regulation that is an important regulator of the apoptotic pathway. During *M. tb* infection, mTOR is activated and consequently downregulates PDCD4, leading to decreased apoptosis. This reveals that utilizing mTOR inhibitors is a potential therapeutic avenue for tuberculosis infection. Glucocorticoids, a steroid that influences glucose metabolism and has immunosuppressive effects, have been identified as a potential mTOR pathway inhibitor. This leads to the upregulation of PDCD4 expression and therefore inhibits *M. tb* proliferation in macrophages. This interplay between PDCD4, glucocorticoids, and mTOR can be a potential marker for future therapies combating *M. tb* infection [58].

By manipulating the host mTOR signaling pathway, *M. tb* can evade the host immune system and disrupt host metabolism to ensure its survival. Understanding these different signaling pathways can lead to novel therapies that may counteract *M. tb* survival tactics.

## 6. Current and Future Therapeutics Targeting OR

### 6.1. Current Therapeutics

Currently, the front-line treatment plan for drug-sensitive strains of *M. tb* is a 6-month-long regimen that involves four different drugs and strict patient compliance to avoid relapse or the development of drug resistance [59]. The treatment plan is divided into two phases: the initial phase and the continuation phase. The initial phase is administered over the course of two months and involves a cocktail of four drugs, namely isoniazid, rifampicin, pyrazinamide, and ethambutol. The continuation phase lasts for four months and only consists of two drugs, namely isoniazid and rifampicin, to kill the dormant bacteria [59]. As previously mentioned, mycolic acids are found in the outer coating of *M. tb* and contribute to their survival in the host by decreasing its permeability to the host’s immune response. The key enzyme in the biosynthetic process of mycolic acids is enoyl-acyl carrier protein reductase, which is inhibited upon activation of the prodrug isoniazid [60]. Rifampin works to target the rpoB gene, which is a gene that controls the earlier steps of gene transcription by binding to the β subunit of RNA polymerase [61]. The bactericidal activity of rifampicin is responsible for shortening the treatment duration and decreasing the number of relapsing TB cases. Although the current medley of drugs used to treat *M. tb* is effective, there is a need for more research and the development of adjunctive therapies or even new novel therapies, as *M. tb* strains are becoming drug resistant, while current therapies are not evolving as quickly.

### 6.2. Repurposing of Existing Drugs

As an effort to combat the increasing number of drug-resistant strains of *M. tb* to the current front-line treatment, many individuals have investigated the repurposing of drugs that are currently on the market by targeting the host response. One host-directed therapy to *M. tb* that has been studied is the enhancement of autophagy via mTOR dependent and independent pathways. Bazedoxifene is typically used as an estrogen receptor modulator but was also shown to enhance autophagy by increasing the formation of autophagosomes and the expression of proteins related to autophagy in macrophages that have been infected. Thus, there is a correlation between treatment with bazedoxifene and the decreased growth of *M. tb*. Further analysis also revealed that the increase in autophagy activity from bazedoxifene was through activation of the AMPK/mTOR signaling pathway [62]. Amoxapine, an anti-depressant, is another drug that was studied and found to enhance the induction of autophagy in an mTOR-dependent pathway [63]. Everolimus, an mTOR inhibitor, is a drug typically used in organ transplants as an immunosuppressant. Preclinical trials have shown that everolimus has the ability to impact host immune response via the mTOR pathway and thus modulate autophagy [64]. In a study conducted with a human granuloma model, everolimus was found to significantly reduce bacterial load, providing itself as a potential treatment to decrease mycobacterial activity by modulating the immune environment within granulomas. Interestingly, they also found that everolimus provided additive efficacy in controlling *M. tb* infection in granulomas when combined with the front-line anti-TB drugs isoniazid and pyrazinamide [65]. The identified benefits of everolimus include decreased dependency on the use of directly observed treatment therapy [33]. Furthermore, Paroha et al. investigated 786 mTOR inhibitors and compared their responses to mycobacterial infection and found that select compounds such as everolimus consistently inhibited the mTOR pathway and had increased autophagy with decreased bacterial load when infected. These findings provide further confirmation on the efficacy of mTOR inhibitors in combating mycobacterial growth, reinforcing their mechanism as a promising treatment strategy in the development of future therapies [58]. While bazedoxifene, amoxapine, and everolimus utilize the mTOR pathway, anticonvulsants such as carbamazepine and valproic acid can enhance autophagy without the mTOR pathway. To decrease bacterial load, carbamazepine and valproic acid stimulate autophagy through the depletion of myo-inositol, which is a cellular metabolite needed for ATP production. Using mouse models infected with a multidrug-resistant strain of *M. tb*, researchers found a decrease in bacterial load, less lung pathology, and enhanced responses from host adaptive immunity when treated with carbamazepine [66]. Although many current drugs seem to play a role in treating *M. tb* through the enhancement of autophagy and increased intracellular bacterial clearance, further clinical trials and research still need to be conducted to validate their efficacy and safety (Table 1).

### 6.3. Potential Therapies to Explore

As lack of compliance, difficulty accessing proper treatment, and poverty contribute to the increasing number of drug-resistant strains of *M. tb*, the need to develop more effective treatment plans continues to increase. With the increasing emergence of drug-resistant strains, there could be a demand for a longer treatment duration with more harmful drugs compared to what is currently on the market, which is why potential therapies are looking to explore host-directed therapies. Precision medicine displays itself in infectious disease by targeting immune cell regulatory pathways as a host-directed therapy. In relation to *M. tb*, exploration with precision medicine through therapeutic pathways would focus on essential core regulatory mechanisms, such as autophagy, or targeting the function of immune cells before they combat infection to improve patient outcomes and improve treatment efficacy [67]. The aim of host-directed therapy is to change the way the host responds to the infection by improving the host’s immune defenses to decrease the duration of treatment or the effect of the infection to the host [68]. The mTOR pathway is regulated by host cell autophagy and plays a critical part in cellular homeostasis and host defense against mycobacteria, making it an excellent target for the development of new therapies. Therefore, further research of this pathway could provide a novel adjunctive therapy to the current TB antibiotics, potentially improving patient outcomes and decreasing drug resistance [75]. Rapamycin used as a therapy to treat *M. tb* only partially inhibits mTORC1, but Li et al. found that the addition of CC214-2, an inhibitor of both mTORC1 and mTORC2, to the current front-line treatment regimen resulted in fewer relapses in C3HeB/FeJ mice compared to the mice treated with rapamycin, indicating that CC214-2 and related mTOR kinase inhibitors could be a potential candidate for host direct therapy [68,69].

Bacillus Calmette–Guérin (BCG) is the only approved TB vaccine to protect against TB. As BCG is an attenuated mutant of *Mycobacterium bovis*, this vaccine does not induce autophagy as much as other strains of mycobacteria. Thus, lower levels of autophagy induction could potentially weaken the adaptive immune responses hosts have in place to control TB, which is why researchers proposed enhancing autophagy and antigen presentation with Mycobacteria smegmatis pre-infection, as it could potentially enhance vaccination protection and has shown to have increased autophagy activation when compared to the BCG alone [70].

As granulomas are a hallmark of *M. tb,* their process of formation involving a variety of immune cells could be a target for developing therapies. Glutathione plays an active role in the function of immune cells, specifically T cells, and participates in the regulation of cytokines, redox activities, and reduction in free radicals. As glutathione is an immunomodulatory antioxidant that stabilizes redox activity, its effects allow cytokines to initiate Th1 type responses and enhance T lymphocytes, and it is therefore reported as an adjunctive therapy that could be used for *M. tb* [71]. This claim is supported, as the depletion of glutathione increased bacterial load and impaired immune cell function while altering cytokine profiles and increasing oxidative stress [72]. To further emphasize the potential of glutathione, mice with supplemented liposomal glutathione have been shown to have increased immune-supportive cytokines in the lungs, reduced *M. tb* burden, and decreased oxidative stress during *M. tb* infection, resulting in improved control of infection in lung granulomas [73]. In individuals with type 2 diabetes, those that were supplemented with liposomal glutathione had significantly decreased the intracellular burden of *M. tb* within in vitro granulomas [74]. With many studies revealing the efficacy of glutathione in conjunction with host immune defense, more therapies aimed to increase glutathione levels could strengthen host immunity and improve patient outcomes in TB treatment (Table 1).

## 7. Conclusions

TB, caused by *M. tb*, proves to be detrimental to human health across the globe and is becoming increasingly difficult to treat with long treatment regimens, limited vaccine efficacy, and emerging antibiotic resistance. This paper focused on reviewing existing literature which studied the effect of mTOR, the PI3K/mTOR/AKT pathway, and autophagy on *M. tb* survival. From our extensive analysis of the pathology of TB, we know the infectious pathogen manipulates cellular machinery in the cell to evade phagosomal elimination, and thus *M. tb* can develop resistance to autophagy. After outlining the mechanism of action of mTOR, the pathways through which it acts, and its various impacts in the body, we found that mTOR plays a critical role in the autophagy process and affects *M. tb* infection and clearance in the host. The mTOR pathway presents itself as a promising target for developing new therapies. Everolimus, rapamycin + CC214-2, and GSH are all current drugs of interest and are being studied from various angles or in combination with other medications against mycobacterial infection. Other avenues such as Chinese herbal plants, baicalin, and BTLA all modulate the PI3K/mTOR/AKT pathway to upregulate autophagy and increase the clearance of mycobacteria from the host. These various therapeutic methods show great promise for the future of *M. tb* treatment; however, there remains a gap in comprehending the full spectrum of the benefits and consequences they can have on the host during *M. tb* infection. We know the interplay between *M. tb* and mTOR during infection is complex, and by understanding this pathway and how they associate with each other in the system, we can learn new targets of study and further develop novel therapies to treat tuberculosis.

## Figures and Tables

**Table 1:** This table highlights the various potential therapies that should be further explored and their targets and mechanisms of action.

Therapy	Target and Mechanism of Action	Outcome of Therapy Use in *M. tb*	Ref
Bazedoxifene	Estrogen receptor modulator, activates AMPK/mTOR signaling	Enhance autophagy through increased autophagosome formation and proteins involved with autophagy	[62]
Amoxapine	Anti-depressant	Enhance autophagy	[63]
Everolimus	mTOR inhibitor	Reduce bacterial load, decrease mycobacterial activity	[64,65]
Carbamazepine and Valproic acid (anticonvulsants)	Depletes myo-inositol	Stimulate autophagy, decrease bacterial load, enhance host adaptive immunity	[66]
Precision Medicine	Target autophagy or immune cell function	Improve host’s immune defense to decrease treatment duration and effect of infection	[67]
Rapamycin + CC214-2	Partial inhibition of mTORC1 (from rapamycin) + inhibition of mTORC1 and mTORC2 (from CC214-2)	Fewer relapses of infection	[68,69]
Mycobacteria smegmatis pre-infection	Potentially enhance vaccination protection of Bacillus Calmette–Guérin (BCG)	Increased autophagy activation	[70]
Glutathione	Initiate Th1 type responses, enhance T lymphocytes	Decrease oxidative stress, improved control of infection in lung granulomas, decrease intracellular burden of *M. tb*	[71,72,73,74]

## Data Availability

No new data were created or analyzed in this study. Data sharing is not applicable to this article.
